# Oxidized LDL and LOX-1 in Experimental Sepsis

**DOI:** 10.1155/2013/761789

**Published:** 2013-08-13

**Authors:** Nadia Al-Banna, Christian Lehmann

**Affiliations:** ^1^Department of Anesthesia, Pain Management and Perioperative Medicine, Dalhousie University Halifax, Nova Scotia, Canada B3H 2Y9; ^2^Department of Pharmacology, Dalhousie University Halifax, Nova Scotia, Canada B3H 2Y9; ^3^Department of Microbiology and Immunology, Dalhousie University Halifax, Nova Scotia, Canada B3H 2Y9; ^4^Department of Anesthesiology and Operative Intensive Care Medicine, Charité, Universitätsmedizin, Berlin, Germany

## Abstract

Oxidized low-density lipoproteins (oxLDL) and the lectin-like oxLDL receptor-1 (LOX-1) are upregulated in inflammation. Because of the importance of inflammation and capillary leakage in the impairment of the microcirculation, which in turn contributes to the development of sepsis and multiorgan failure, the role of oxidized LDL and LOX-1 as players of intestinal inflammation is of great interest. In fact, the blockade of LOX-1 during experimental endotoxemia was effective in reducing leukocyte activation. There are several mechanisms by which oxLDL can participate in local and systemic inflammation, including cell proliferation, apoptosis, capillary perfusion, leukocyte-endothelial cell interactions, and endothelial activation. This review highlights the evidence relating oxLDL and LOX-1 to proinflammatory disease mechanisms. We also indicate situations when oxLDL, because of exposure time, dose, or degree of oxidization, is involved in disease resolution. Modulation of LOX-1 response could be utilized for the treatment of local and systemic inflammation, but the successful use of this target requires further understanding of its broad effects.

## 1. Introduction

Recently, the contribution of oxidized LDL (oxLDL) to leukocyte activation and microvascular perfusion disturbances in experimental endotoxemia has been reported [1]. It is thought that oxidized LDL and LOX-1 may play a role in the increased inflammation and capillary leakage, two factors which induce disturbances in the microcirculation. Because of the importance of impaired microcirculation in the development of sepsis, and the ensuing organ failure, it is important to consider oxidized LDL and LOX-1 as players of intestinal inflammation. In this review, our goal is to introduce the literature on oxLDL in local and systemic inflammation and its relation to sepsis development. We also discuss the variety of mechanisms by which oxLDL can participate in inflammation, including cell proliferation/apoptosis, capillary perfusion status, leukocyte-endothelial cell interaction, neutrophil recruitment, leukocyte activation, and the endothelial cell response. While considering its potential for promoting proinflammatory disease mechanisms, we also highlight situations when it can be involved in the disease resolution. It seems obvious that this axis should be utilized for the treatment of intestinal inflammation and sepsis, but significant literature gaps need to be addressed beforehand.

## 2. Lectin-Like oxLDL Receptor

The lectin-like oxLDL receptor-1 (LOX-1) binds the protein moiety of oxLDL [2]. This receptor was first studied in vascular endothelial cells [3], and it was later shown to be expressed in human intestinal cell lines [4], endothelial cells, macrophages, and smooth muscle cells [5]. Several factors are known to upregulate LOX-1, including endotoxin (lipopolysaccharide; LPS), shear stress and oxidative stress [5], and the presence of oxLDL itself [4]. In addition,* in vitro *cultured smooth muscle cells were shown to upregulate the expression of LOX-1 in the presence of inflammatory cytokines, including IL-1alpha, IL-1beta, and TNF-alpha [6].

The oxidation of LDLs can occur by activated leukocytes, whether activated polymorphonuclear cells [7], macrophages, or neutrophils [8]. In the presence of oxLDL, lipid body formation is enhanced in leukocytes, as shown for mouse peritoneal macrophages after 1 hour of *in vitro* culture [9]. This was also shown *in vivo*; the intraperitoneal injection of phospholipid fractions of oxLDL into mice resulted in the development of lipid body formation within 3 hours [9]. In addition, the upregulation of LOX-1 on intestinal cell lines was associated with the enhanced uptake of other factors (e.g., pancreatic bile salt-dependent lipase) into the cells [4], reflecting the overall lipid dysfunction that occurs as part of the inflammatory process.

## 3. LOX-1 in Experimental Sepsis

Oxidation of LDL was illustrated in animals with endotoxemia. In fact, LPS-injected hamsters were found to have higher levels of oxidized fatty acids in serum, namely, a 4-to 6-fold increase in the levels of conjugated diene and lipid hydroperoxide in LDL fraction and 17-fold increase in LDL content of lysophosphatidylcholine. In our own study, we reported the increased expression of LOX-1 in the intestinal tissues of animals with endotoxemia, both at the mRNA and the protein level [1]. These studies indicate the oxidation of LDL after LPS injection, as would occur during acute infection and during inflammatory conditions. 

 Mice that lacked the expression of LOX-1 (i.e., LOX-1^−/−^ mice) were found to have improved survival in a cecal ligation and puncture (CLP) sepsis model. LOX-1^−/−^ mice also had lower inflammatory response to CLP, in terms of pro-inflammatory cytokine levels (e.g., TNF-alpha) in the serum and the extent of lung damage (e.g., edema). These mice were also able to clear the bacteria from peritoneum, blood, and lungs more than wild mice [10]. The contribution of LOX-1 in the development of lung tissue damage in sepsis was also observed in other models of sepsis. Intraperitoneal injection of LPS increased LOX-1 expression in mouse lung. When LOX-1 is blocked in mice with endotoxemia, the lung tissue damage was significantly reduced, as indicated by the reduced numbers of neutrophils, lower expression of adhesion molecule (intracellular adhesion molecule-1; ICAM-1), and reduced activation of transcription factor NF-*κ*B in the lungs [11]. These effects may explain why animals with endotoxemia pretreated with anti-LOX-1 antibody had partially recovered from the LPS-induced leukopenia and showed improved survival [12].

OxLDL and LOX-1 are also involved in several cardiovascular diseases, for example, atherosclerosis [13] and autoimmune diseases, for example, antiphospholipid syndrome [14]. For instance, peripheral blood mononuclear cells (PBMCs) from patients with antiphospholipid syndrome were shown to produce more pro-inflammatory cytokines (e.g., IFN-gamma and IL-2) in the presence of oxLDL [14]. Since oxLDL can be formed by leukocytes themselves [7, 8], this mechanism represents a vicious cycle that augments the inflammatory process in several diseases. 

## 4. Proposed Mechanisms

**Figure 1:**
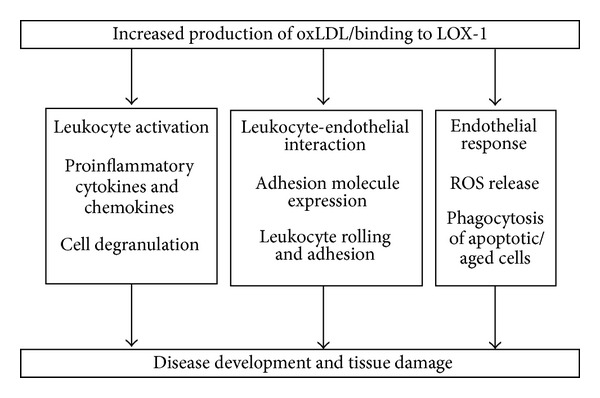
Schematic presentation of selected cellular effects of oxidized LDL (oxLDL) and/or Lectin-like oxLDL receptor (LOX-1) activation in systemic inflammation.

Because of the broad expression of LOX-1 on several cells [3, 5] and the ability of macrophages, neutrophil, and other immune cells to oxidize LDL [7, 8], there are several possible ways by which oxLDL can influence the inflammatory process. This is summarized in Figure 1.

### 4.1. Cell Proliferation/Apoptosis

OxLDL and LOX-1 has been associated with cell death. The growth of *in vitro* activated blood lymphocytes was partially inhibited by treatment of oxLDL after 48 h [15]. OxLDL treated T cell lines [15] and cardiomyocytes [16] were shown to undergo apoptosis. *In vitro* studies have suggested that cell apoptosis is enhanced by the presence of other inflammatory signals, for example, the expression of chemokine receptors, and the production of cytokines TNF-alpha and IL-1beta. This explains why these effects were abolished by the blockade of LOX-1 [16].

It is important to note that the ability of oxLDL to induce apoptosis is influenced by the degree of oxidation, dose of oxLDL, and the exposure time. Apoptosis was induced in macrophage cell lines by incubation with extensively oxidized LDL (at 100 mg/mL) or with a higher dose (200 mg/mL) of light or extensively oxidized LDL [17]. In contrast, macrophage activation and proliferation were induced by low dose (100 mg/mL) of the lightly oxidized LDL. Interestingly, the use of shorter incubation time induced cell proliferation, even if the LDL was highly oxidized LDL and at a lower dose (100 mg/mL) [17].

### 4.2. Capillary Perfusion

Studies examined whether the microhemodynamic parameters are modified by oxLDL [18] or LOX-1 expression [1]. In hamsters treated with oxLDL or endotoxemic rats treated with LOX-1 blockade, the capillary microperfusion was not affected [1, 18]. When endothelial-dependent vasodilation in isolated microvessels exposed to oxLDL was observed, the effect was related to the oxidative stress, since it was restored by incubation with oxygen radical scavengers [19]. 

### 4.3. Leukocyte-Endothelial Cell Interactions

#### 4.3.1. Expression of Adhesion Molecules on Endothelial Cells

There is an increased expression of adhesion molecules on endothelial cells in the presence of oxLDL or in association with LOX-1 expression. OxLDL treatment was shown to stimulate the adherence of THP-1 (a human acute monocytic leukemia cell line) to human umbilical vein endothelial cells, in conjunction with an increased expression of LOX-1 and several adhesion molecules (namely, Intracellular cell adhesion molecule-1 (ICAM-1), Vascular cell adhesion molecule-1 (VCAM-1) and E-selectin) on the endothelial cells [20].

In another study, both xanthoma tissue-modified LDL (x-LDL) and copper sulfate oxidized-LDL (Cu-LDL) were able to induce the adhesion of monocyte leukemic cell lines (U937) to human dermal microvascular endothelial cells (HDMEC), but this was related to upregulation in the expression of different adhesion molecules depending on the kind of oxLDL. For instance, x-LDL induced upregulation of VCAM-1 and E-selectin, and Cu-LDL upregulated VCAM-1, E-selectin, and ICAM-1 on endothelial cells [21]. This mechanism may be responsible for the effects of other inflammatory mediators. For example, C-reactive protein was shown to induce the expression of ICAM-1 and VCAM-1 on human endothelial cells, but this was inhibited when LOX-1 was blocked (using LOX-1 small-interfering RNA) [22].

#### 4.3.2. Leukocyte Rolling and Adhesion

The interaction between leukocytes and endothelium in the presence of oxLDL or LOX-1 has been studied both *in vitro* and *in vivo*. Surface coating with LOX-1 was sufficient to induce the adhesion of polymorphonuclear cells (PMNs) under physiologic shear conditions [12]. Platelets were also shown to bind to endothelium using the LOX-1 and oxLDL interaction. In fact, the negative phospholipids present on surface of platelets were suggested to be ligands for LOX-1. Therefore, the presence of oxLDL on endothelium was shown to enhance platelet aggregation and to lead to an induction of endothelin-1 in the endothelial cells (a sign of endothelial dysfunction) [23]. When the interaction of copper-oxidized LDL treated endothelium with flowing erythrocytes and platelets was examined using intravital microscopy, there was a transient increase in the number of platelet-endothelial cell adhesions. It is important to note that this effect was inhibited by the administration superoxide dismutase and catalase, suggesting the participation of oxygen-derived free radicals in the oxLDL-induced effects [24].

Several studies utilized oxLDL to induce leukocyte rolling and/or adhesion on endothelium. Local intra-arterial infusion of oxLDL was shown to increase the leukocyte rolling across rat mesenteric venules [25]. It was also reported to promote the adhesion of neutrophils and leukocytes to endothelium *in vitro*, in a manner that was dependent on the degree of lipid peroxidation. Local intra-arterial infusion of oxLDL elicited significant increases in leukocyte adherence and albumin leakage in rat mesenteric venules, without affecting the venular shear rate [25, 26].

The administration of oxLDL in rats was shown to increase leukocyte-endothelial interactions by increasing the number of rolling cells and reducing their rolling velocity [27]. It is interesting to note that these effects were not observed in pregnant rats [27]. Using another model, the skinfold chamber model in hamsters, oxLDL administration induced leukocyte rolling and adhesion to the endothelium of venules and arterioles [18]. This may be related to the action of PAF on leukocyte adhesion, as shown by the ability of PAF receptor antagonism to attenuate oxLDL-induced leukocyte adhesion *in vivo* [18]. In addition, they may be related to the action of inflammatory mediators (e.g., leukotrienes) and oxidative stress determinants (e.g., superoxide radicals).

The role of LOX-1 was specifically tested in the context of endotoxemia. Our group has modeled the increased adhesion of leukocyte to intestinal venules in animals with endotoxemia. Blocking LOX-1 had an inhibitory effect on leukocyte adhesion. Using intravital microscopy, we demonstrated that blocking LOX-1 was sufficient to reverse the LPS-induced increase in leukocyte adhesion to intestinal venules [1]. Likewise, Honjo et al. [12] demonstrated that LOX-1 blockade suppresses leukocyte infiltration and protein exudation in animals with endotoxin-induced uveitis. In addition, the retinal microvessels appear to have reduced interaction with leukocytes in animals with endotoxemia after LOX-1 blockade, with regard to the reduced number of rolling cells and increased velocity of rolling. This study therefore suggested LOX-1 as a vascular tethering ligand specifically [12].

### 4.4. Neutrophil Recruitment

Since LOX-1 is expressed in neutrophils, the function of LOX-1 was important to examine. LOX-1 deletion was found to increase the activation and recruitment of neutrophils to infection sites, for example, peritoneum in CLP-induced sepsis [10]. The mechanism of this phenotype is not clear to us at this point. 

### 4.5. Leukocyte Activation

#### 4.5.1. Inflammatory Signaling Pathways

OxLDL can modulate the NF-*κ*B pathway. Treatment of human PBMCs with oxLDL leads to an increased NF-*κ*B induction (p65 nuclear translocation) [28], and the same was reported for human umbilical vein endothelial cells (HUVECs) [20] and aortic endothelial cells that express LOX-1 [29]. This effect was correlated with an increased production of IL-6 and expression of toll-like receptor (TLR)-2 and TLR-4 in oxLDL-treated PBMCs [28], and an increased level of reactive oxygen species [29].

Moreover, the treatment of minimally oxidized LDL on LPS-activated macrophages was reported to upregulate their NF-*κ*B and AP-1 pathways, in conjunction with an increased expression of inflammatory chemokines [30]. In contrast, oxLDL can delay the activation of NF-*κ*B in LPS-treated peritoneal macrophages [31], and therefore it may have immunosuppressive effects during immune response. These contrasting reports are likely due to the differences in concentrations of ox-LDL [32] or the degree of oxidation of the LDL used [30]. 

It should be noted that the effect of oxLDL on the activation of signaling pathways can be cell specific. For instance, the addition of copper oxidized-LDL to *in vitro* cultures of vascular smooth muscle cells and human monocyte-derived macrophages was shown to induce the phosphorylation of MAP kinase, but this was not the case for bovine endothelial cells [33]. 

#### 4.5.2. Cytokine and Chemokine Production

Similar to what was reported on the effect of oxLDL in the activation or inhibition of inflammatory signaling pathway, its impact on cytokine production also varies. On one hand, the treatment of oxidized LDL can increase the production of IL-8 from freshly isolated monocytes and monocytic THP-1 cell lines [34], HUVECs [20], and LOX-1 overexpressing human aortic endothelial cell line [35]. OxLDL administration was also shown to induce the production of IL-6 from HUVECs [20], chemokines CXCL2 and CXCL3 from LOX-1 overexpressing human aortic endothelial cell line [35], MCP-1 from human articular chondrocytes [36], and the intestinal tissue of animals with endotoxemia [1]. In one study, the capacity of oxLDL to induce the production of IFN-*γ* and TNF-alpha from PBMCs was suggested to be related to the PAF-like lipids in oxLDL, since this function was at least partially inhibited by PAF-receptor antagonist [37]. In striking contrast, oxLDL was reported to inhibit the ability of LPS-activated peritoneal macrophages to produce a number of cytokines and chemokines [31].

#### 4.5.3. Mast Cell Activation/Degranulation

It is interesting to note that oxLDL can induce mast cell degranulation, as shown when oxLDL was locally infused in rat mesentery [26]. This effect was suggested to promote leukocyte-endothelial cell adhesion and albumin leakage responses to copper-oxidized LDL, that were inhibited by pretreatment with the mast cell-stabilizing agents [26]. It is worthwhile to consider the vast breadth of immune cells that can be affected by oxLDLs; it is important to note that the variation in the effect of oxLDL/LOX-1 on cytokine production and inflammatory signaling pathways that were mentioned above may also impact the cell activation status. 

### 4.6. Endothelial Cell Response

#### 4.6.1. Reactive Oxygen Species (ROS) and Free Radical Production

The generation of ROS, inducible NO synthase, NO, and oxygen radicals by HUVECs [20] and aortic endothelial cells [38] was found to increase after oxLDL treatment, even as early as 30 seconds of incubation [29]. This is important, since the blockade of superoxides, using ellagic acid, in oxLDL treated HUVECs inhibited many of the downstream effects of LOX-1, including the phosphorylation of MAPK and NF-*κ*B pathways, the production of cytokines, and expression of adhesion molecules to HUVECs [20].

#### 4.6.2. Matrix Metalloproteinase (MMP)

When human endothelial cells were cultured *in vitro* and treated with oxLDL, they increased the mRNA and protein level of membrane type 1-matrix metalloproteinase (MT1-MMP), an enzyme, that is known to promote the activation of matrix degradation (by activating pro-MMP2). This effect occurred within 6 hours of exposure and was even further enhanced when cells were treated with TNF and oxLDL in combination [39].

#### 4.6.3. Phagocytosis of Apoptotic/Aged Cells

The expression of LOX-1 by endothelial cells and Chinese hamster ovary cells was associated to their ability to bind aged RBC and apoptotic cells. As such, the treatment of mAB to LOX-1 was found to inhibit the binding of aged RBC to these cells. In fact, the study by Oka et al. suggested that the ability of oxLDL to bind to aged RBCs was due to the PS part of the surface of apoptotic cells, and therefore this effect increases coagulation and inhibits the removal of apoptotic cells [40]. The function of LOX-1 as a scavenger receptor may extend to several other usages. In fact, IFN-alpha-activated dendritic cells were found to upregulate LOX-1, where it appears to participate not only in the uptake of apoptotic cells but also for CD8 T cell priming [41].

## 5. Concluding Remarks

 Sepsis and intestinal inflammation involve significant changes in oxLDL levels and LOX-1 expression. Studies have demonstrated the efficacy of blocking or knocking out LOX-1 in decreasing the inflammatory process and tissue damage that occur in models of sepsis. Oxidized LDL is produced by a variety of cells, and it can bind to intestinal endothelial cells, as shown by cultured *in vitro* and *in vivo* studies. Research has provided evidence for a wide range of effectors that are modulated by the upregulation of oxLDL and the activation of LOX-1 (summarized in Figure 1). These include enhancing the leukocyte-endothelial interactions, whether by increasing the expression of adhesion molecules on endothelial cells or by increasing the rolling and adherence of leukocytes to the microcirculation. They were also shown to activate leukocytes, evident as activation of NF-*κ*B pathway, production of cytokines and chemokines, and degranulation. In addition to these, oxLDL and LOX-1 can influence the endothelial cell responses, in terms of ROS production, MMP, levels and the phagocytosis of apoptotic cells. 

This review summarizes the significant efforts that aim to understand the breadth of effects induced by oxLDL and LOX-1. However, it is important to note that the contribution of oxLDL to the development of sepsis is a relatively newer domain, in comparison to the literature available on the lipid dysfunction in cardiovascular diseases. There is still a significant need for examining this key mediator in more detail, in order to be able to target it specifically to control the development of sepsis. Challenges include the fact that, under certain situations, oxLDL was shown to have some anti-inflammatory roles. Thus, it is crucial to consider the dose, time of exposure, and the degree of oxidation when studying this mediator. In the future, the specific ability to control these elements will determine the effectiveness of using LOX-1 blockade strategies to alleviate the inflammatory response in sepsis.
